# Acute renal cortical necrosis following postpartum hemorrhage – successful discontinuation of peritoneal dialysis: A case report 

**DOI:** 10.5414/CNCS111741

**Published:** 2025-12-19

**Authors:** Chiho Fukushima, Kenichi Koga, Masako Hasebe, Chiaki Omiya, Keisuke Nishioka, Kensei Yahata

**Affiliations:** Department of Nephrology, Osaka Red Cross Hospital, Osaka, Japan

**Keywords:** renal cortical necrosis, pregnancy-related acute kidney injury, peritoneal dialysis, renal biopsy

## Abstract

Renal cortical necrosis (RCN) is a rare but severe cause of acute kidney injury primarily observed in obstetric complications. We herein present a case of RCN in a 32-year-old Mongolian primigravida transferred to our hospital with uncontrolled massive postpartum hemorrhage. Contrast-enhanced computed tomography revealed ongoing uterine hemorrhage, prompting endovascular intervention for control. It also showed the loss of cortical perfusion in the kidneys, while preserving medullary blood flow, which is consistent with RCN. The patient remained anuric from transfer and required continuous hemodiafiltration followed by intermittent hemodialysis. Renal biopsy on day 23 revealed coagulative necrosis in glomeruli and tubules in the outer cortex, consistent with RCN, while glomeruli in the deeper cortex were spared. Glomeruli in the outer cortex displayed glomerular paralysis. The patient transitioned to peritoneal dialysis (PD) to facilitate infant care at home. Over 6 months, renal function improved, allowing dialysis discontinuation. Four years post-discharge, she remains free of renal replacement therapy, with serum creatinine of 2.67 mg/dL. The present case highlights the potential for gradual renal function improvement in RCN through the recovery of residual nephrons. PD may be a promising modality for patients with RCN.

## Introduction 

Renal cortical necrosis (RCN) is a rare cause of acute kidney injury (AKI). It typically occurs in pregnant women and is often associated with severe complications, such as massive obstetric bleeding [1]. RCN presents a significant clinical challenge in developing countries where the management of obstetric hemorrhage is hindered by inadequate healthcare facilities [1, 2]. However, RCN is still encountered in developed countries, accounting for 1% to 2% of all causes of AKI [3]. RCN mostly presents with anuria or severe oliguria, often necessitating renal replacement therapy (RRT) [1]. While some patients may discontinue RRT [4], effective treatments and accurate predictions of renal outcomes have yet to be fully established. We herein present a case of obstetric RCN that initially required acute hemodialysis (HD), followed by maintenance peritoneal dialysis (PD) for 6 months, ultimately leading to the successful withdrawal of RRT. 

## Case report 

A 32-year-old Mongolian female, with unremarkable medical and family histories, presented with the onset of proteinuria at 38 weeks gestation during her first pregnancy. Urinalysis at 39 weeks gestation revealed significant proteinuria (3+; urine protein/creatinine ratio of 3.06 g/gCr), raising suspicion of pre-eclampsia, and she was admitted to a different medical facility. Cesarean section was performed at 40 weeks, and she developed hypertension with blood pressure exceeding 160/80 mmHg following the procedure. The patient had massive intraoperative and postoperative bleeding, with blood losses of 600 and 1,880 mL, respectively, and was administered a fluid infusion of 2,000 mL. She was transferred to our hospital due to uncontrollable bleeding. 

**Figure 1.**
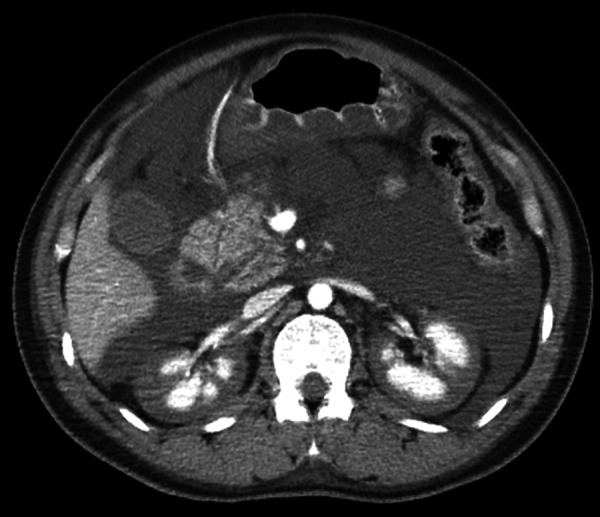
Abdominal contrast-enhanced computed tomography findings. The renal cortex displays a non-enhancing pattern, while the medulla demonstrates enhancement, which is indicative of the reverse rim sign consistent with renal cortical necrosis.

Upon admission, blood pressure was 102/64 mmHg, pulse rate 130 beats/min, and body temperature 36.5 °C, accompanied by drowsiness and conjunctival pallor. Laboratory findings upon admission included severe anemia (hemoglobin: 4.5 g/dL), leukocytosis (23,130/μL), thrombocytopenia (platelet count: 10.1 × 10^4^/μL), impaired renal function (serum creatinine: 1.64 mg/dL), hyponatremia (Na: 126 mEq/L), and hyperkalemia (K: 5.2 mEq/L) (Table 1). A further evaluation revealed evidence of coagulopathy, with a prolonged prothrombin time (PT/INR: 1.50), low fibrinogen levels (108 mg/dL), and significantly increased fibrin degradation products (104 μg/mL). These findings led to a diagnosis of disseminated intravascular coagulation (DIC), likely due to fibrinogen consumption from massive bleeding. Liver function tests were within the normal ranges. In the emergency room, the patient’s bleeding persisted, and she received 11 units of red blood cells, 16 units of fresh frozen plasma, and 20 units of platelets. Abdominal contrast-enhanced computed tomography was performed to identify the source of uterine bleeding and revealed contrast extravasation from the uterine wall into the uterine cavity. Additionally, the renal cortex displayed a non-enhancing pattern, while the medulla showed enhancement, which is indicative of the reverse rim sign consistent with RCN (Figure 1). 

**Figure 2.**
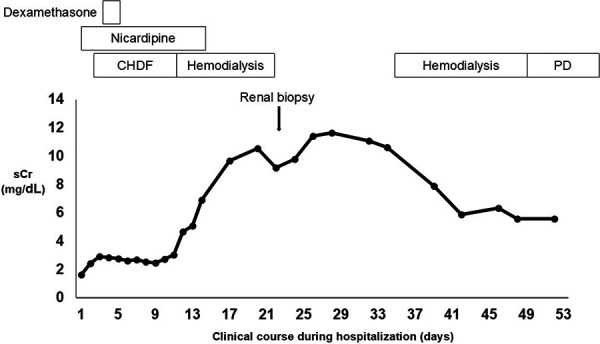
Clinical course during hospitalization. sCr = serum creatinine; CHDF = continuous hemodiafiltration; PD = peritoneal dialysis.

**Figure 3.**
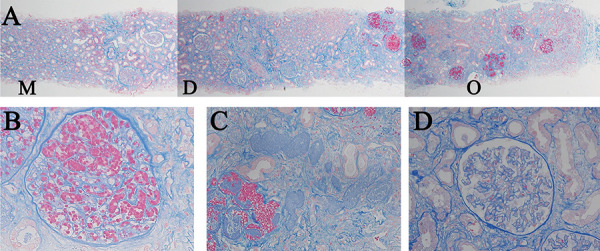
Renal biopsy findings. A: Low-power magnification image (Masson trichrome staining; original magnification × 40). M = medulla; D = deeper cortex; O = outer cortex, B: High-power magnification image of the outer cortex showing glomeruli with glomerular paralysis and coagulative necrosis (Masson trichrome staining; original magnification × 200), C: High-power magnification image of the outer cortex with tubules showing coagulative necrosis with the loss of normal cytologic features (Masson trichrome staining; original magnification × 200), D: High-power magnification image of the deeper cortex (Masson trichrome staining; original magnification × 200). The glomeruli and tubules were spared from necrosis; however, ischemic changes were observed.

**Figure 4.**
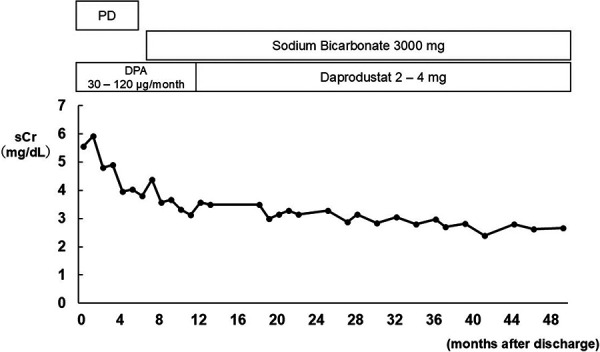
Post-discharge clinical course. sCr = serum creatinine; PD = peritoneal dialysis; DPA = darbepoetin α.

Due to uncontrollable uterine bleeding, an endovascular intervention was performed to control hemorrhage, successfully achieving hemostasis. Since the patient remained anuric after transfer, continuous hemodiafiltration (CHDF) was initiated in the intensive care unit (ICU) (Figure 2). The patient developed persistent thrombocytopenia with a platelet count below 50,000/μL, elevated liver enzymes (with maximal aspartate aminotransferase and alanine aminotransferase levels reaching 362 and 110 U/L, respectively, on the 3^rd^ day), and a decreased haptoglobin level of 36 mg/dL (type 2-1, normal range: 66 – 218 mg/dL). These findings led to a diagnosis of hemolysis, elevated liver enzymes, and low platelets (HELLP) syndrome. In addition to pre-eclampsia and HELLP syndrome, which are major contributors to thrombotic microangiopathy (TMA) during pregnancy, other potential causes of TMA were investigated. The patient did not have diarrhea, and stool cultures did not detect Shiga toxin-producing *Escherichia coli* (STEC), ruling out STEC-associated hemolytic uremic syndrome. A significant decrease in a disintegrin and metalloproteinase with thrombospondin type 1 motif, member 13 (ADAMTS13) activity was not observed, and no ADAMTS13 inhibitors were detected, ruling out thrombotic thrombocytopenic purpura. Genetic testing for abnormalities associated with atypical HUS was submitted, and later results showed no genetic mutations. Her liver enzyme levels and thrombocytopenia gradually improved with antihypertensive therapy using a continuous infusion of nicardipine, along with a 2-day course of dexamethasone for HELLP syndrome. The patient was discharged from the ICU on the 11^th^ day, transitioning from CHDF to intermittent HD. Renal biopsy on the 23^rd^ day confirmed the diagnosis, revealing coagulative necrosis of glomeruli and tubules in the outer cortex (superficial cortical region), consistent with RCN (Figure 3A). Superficial glomeruli in the outer cortex, which had undergone coagulative necrosis, displayed glomerular paralysis with abundant erythrocytes in dilated glomerular capillaries (Figure 3B), and tubules in the same region showed coagulative necrosis with the loss of normal cytologic features (Figure 3C). Glomeruli and tubules in the deeper cortex (juxtamedullary region) were spared from necrosis, showing a preserved cellular structure (Figure 3D). There was no evidence of glomerulonephritis, although ischemic changes were observed. No apparent thrombi were detected in the small arteries or arcuate arteries in the regions spared from necrosis; however, it was difficult to assess the presence of thrombi in vessels within necrotic areas. Immunofluorescence did not reveal any significant deposits. An electron microscopy sample included only sclerotic glomeruli, making further evaluations unfeasible. She discontinued hemodialysis on the 22^nd^ day after admission but resumed it on the 35^th^ day due to persistent oliguria and solute retention. The patient selected PD as maintenance dialysis, which may be performed at home, in order to care for her child. She began PD on the 48^th^ day and was discharged on the 53^rd^ day. Since her serum creatinine gradually decreased, the patient successfully discontinued dialysis 6 months after discharge, with serum creatinine of 4.39 mg/dL (Figure 4). Four years post-discharge, the patient remains free of RRT, with serum creatinine of 2.67 mg/dL, while receiving treatment for metabolic acidosis and renal anemia. 

## Discussion 

RCN is characterized by cortical necrosis sparing the medulla, with coagulative necrosis and a preserved tubular and glomerular architecture, despite the loss of normal cytology [5]. Pathological studies on RCN in patients described necrotic lesions as either diffuse (complete) or patchy (incomplete), with the cortex not being uniformly necrotized in the latter [4, 6, 7]. The present case exhibited RCN of varying severity, with greater involvement in the outer cortex and milder injury in the deeper cortex. This observation aligns with previous findings of non-necrotized lesions being more likely in the deeper cortex [6]. These differences between the outer and deeper cortices suggest that the distinct characteristics of superficial nephrons (SNs) in the outer cortex and juxtamedullary nephrons (JNs) in the deeper cortex contribute to its pathogenesis. SNs and JNs are primarily perfused by capillaries originating from superficial and juxtamedullary glomeruli, respectively [8]. SNs experience a significantly greater reduction in blood supply than JNs due to angiotensin II-induced vasoconstriction during hypoperfusion [9, 10], which may lead to notably more severe ischemic injuries in SNs. Research from as early as the mid-20th century showed the involvement of vasoconstrictors, such as serotonin and oxytocin, in the development of RCN using animal models [11, 12]. 

In the present case, an important morphological difference was observed between glomeruli in the outer cortex and those in the deeper cortex. Necrotized glomeruli in the outer cortex displayed glomerular paralysis. In contrast, glomeruli in the deeper cortex, which were spared from necrosis, largely maintained a normal morphology. These differences in glomerular lesions between the outer and deeper cortices have not been well documented. Glomerular paralysis, marked by congestion and erythrocyte-filled dilated capillaries, indicates severe endothelial damage, as observed in TMA and DIC [13]. RCN is often accompanied by TMA and DIC [5], and glomerular paralysis may occur in the early stages of RCN [14]. Our case presented with proteinuria and hypertension before delivery, suggesting the onset of pre-eclampsia preceding the onset of RCN. Additionally, DIC developed during obstetric bleeding in the present case. We speculated that glomerular endothelial injury from pre-eclampsia and DIC aggravated the severity of RCN in the present case because SNs are perfused by peritubular capillaries that branch off from the efferent arterioles of superficial glomeruli [8]. These findings suggest that glomerular and post-glomerular hypoperfusion play an important role in the pathogenesis of RCN. However, it is important to note that renal biopsy was performed on the 23^rd^ day, by which time histological changes, such as the recovery of endothelial injury, dissolution of thrombi, or tubular injury, may have partially occurred. In this context, although the difference in morphological changes between the outer and deeper cortices were notable, it remains unclear whether they truly reflect the acute phase of injury. This delay in timing represents an important limitation; however, early renal biopsy in RCN cases may be challenging due to the poor overall condition of patients and associated risks, such as thrombocytopenia and severe anemia. 

The present case developed severe AKI requiring maintenance dialysis, but ultimately became independent of dialysis. Previous case series reported that a substantial percentage of patients with RCN were able to discontinue dialysis, particularly those with patchy RCN [1, 4, 6, 7]. Chugh et al. [7] showed that of the 44 patients who survived the initial illness, 19 became independent of dialysis within 3 months of onset, while 25 continued to require dialysis. One of these patients showed continuous improvement in renal function over 2 years. These findings indicate that surviving nephrons spared from necrosis gradually recover from ischemic injuries and compensate for lost parenchymal function. The degree of renal functional recovery after RCN depends on the amount of viable cortical tissue following the initial insult [6]. Our patient selected PD as maintenance dialysis because it was compatible with her lifestyle, facilitating infant care. Furthermore, PD is generally considered to be associated with the better preservation of residual kidney function than HD [15]. PD was selected in the present case, with the expectation of preserving residual renal function and urine volume during the anticipated histological and functional recovery of nephrons. Renal function gradually improved, and the patient successfully discontinued PD ~ 6 months post-onset. Furthermore, renal function gradually recovered over a 4-year observation period. 

To examine the impact of the dialysis modality (PD or HD) on renal outcomes, we reviewed obstetric RCN cases in which dialysis was continued for more than 1 month [16, 17, 18, 19, 20, 21] (Table 2). This review included 3 cases of PD (cases 1, 3, and 4); however, cases 1 and 3 received intermittent PD, which markedly differs from contemporary PD practices, making direct comparisons challenging. Case 4 received PD, successfully recovered from dialysis by day 44, and was discharged with a serum creatinine level of 3.2 mg/dL. The patient remained independent of dialysis with chronic kidney disease and was lost to follow-up ~ 1 year after discharge. More recovery cases have been reported in HD patients, likely due to its more frequent use for AKI. Cases 5, 7, and 11 discontinued HD after prolonged treatment durations of 210 days, 120 days, and ~ 3 years, respectively, suggesting that even with HD, recovery is possible after extended periods of dialysis if residual kidney function is maintained. Cases 2 and 11 recovered from HD after 43 days and 3 years, respectively, but required the re-initiation of dialysis 15 and 18 months later, underscoring the importance of preserving kidney function during and after dialysis. Although it remains unclear whether HD or PD is superior in terms of renal outcomes due to the small number of reported cases, our review suggests that recovery is possible with either modality. Accordingly, strategies to support kidney function and to remain attentive to the possibility of dialysis discontinuation need to be emphasized regardless of the dialysis modality used. 

In summary, we herein presented a case of obstetric RCN with interesting renal biopsy findings, highlighting variability in severity between the outer and deeper cortices. Even after the initiation of dialysis, the potential for discontinuation needs to be carefully considered. Although further studies are needed, PD has potential as one of the modalities for RRT in patients with RCN. 

## Authors’ contributions 

K.K. conceived the original idea of the study. C.F. drafted the manuscript with support from K.K., M.H., K.N., and K.Y. All authors contributed to the clinical management of the patient, discussed the results, and reviewed the manuscript. 

## Funding 

There was no funding for this report. 

## Conflict of interest 

The authors have no conflict of interest to declare. 

**Table 1.** Table 1. Laboratory findings on admission.

**Laboratory test**	**Results (normal range)**
Peripheral blood	
White blood cell count (/μL)	23,130 (3,300 – 8,600)
Red blood cell count (×10^4^/μL)	165 (386 – 492)
Schistocytes (%)	1 (< 1)
Hemoglobin (g/dL)	4.5 (11.6 – 14.8)
Platelet count (×10^4^/μL)	10.1 (15.8 – 34.8)
Blood chemistry	
Total protein (g/dL)	3.0 (6.6 – 8.1)
Albumin (g/dL)	1.5 (4.1 –5.1)
Aspartate aminotransferase (U/L)	29 (13 – 30)
Alanine aminotransferase (U/L)	8 (7 – 23)
Lactate dehydrogenase (U/L)	494 (124 –222)
Total bilirubin (mg/dL)	0.8 (0.4 – 1.5)
Uric acid (mg/dL)	5.5 (2.6 – 5.5)
Blood urea nitrogen (mg/dL)	10.9 (8 – 20)
Creatinine (mg/dL)	1.64 (0.46 – 0.79)
Sodium (mEq/L)	126 (138 – 145)
Potassium (mEq/L)	5.2 (3.6 – 4.8)
Chloride (mEq/L)	101 (101 – 108)
Coagulation	
PT/INR	1.50
Activated partial thromboplastin time (s)	33.0 (23.4 – 38.0)
Fibrinogen (mg/dL)	108 (200 – 400)
Fibrin degradation products (μg/mL)	104 (0 – 5)
Serology	
C-reactive protein (mg/dL)	0.18 (0 – 0.14)
Immunoglobulin G (mg/dL)	503 (861 – 1747)
Immunoglobulin A (mg/dL)	156 (93 – 393)
Immunoglobulin M (mg/dL)	49 (50 – 269)
Complement component 3 (mg/dL)	70 (83 – 160)
Complement component 4 (mg/dL)	18.1 (13 – 35)
50% hemolytic complement activity (U/mL)	59.7 (29 – 47)
Anti-nuclear antibody	< 40 (< 40)
Anti-cardiolipin antibody	≤ 8 (≤ 8)
Haptoglobin (type 2-1) (mg/dL)	36 (66 – 218)
ADAMTS13 activity (%)	77 (> 0.78)
ADAMTS13 inhibitor (BU/mL)	< 0.5 (< 0.5)
Direct Coombs	Negative
Urinalysis	
Occult blood	3+
Protein (g/gCr)	20.73
N-acetyl-D-glucosaminidase (U/gCr)	2.1 (0.9 – 2.4)
β-2-microglobulin (μg/gCr)	1,685 (4 – 180)
Sediment	
Red blood cells (/high-power field)	≥ 100 (isomorphic)

PT/INR = prothrombin time/international normalized ratio; ADAMS13 = a disintegrin-like and metalloprotease with thrombospondin type 1 motif 13.

**Table 2.** Clinical characteristics of reported cases of obstetric-associated RCN with recovery from dialysis.

**Case No.**	**Authors**	**Year**	**Age**	**Pregnancy disorders**	**Types of dialysis**	**Time on dialysis**	**sCr or eGFR ** **at the last report**
1	Riff DP [16]	1967	26	PE, placental abruption	PD	49 d	sCr 13
2	Walls J [17]	1968	22	PE, placental abruption	HD	43 d	Re-initiated dialysis*
3	Deutsch V [18]	1971	40	PE, placental abruption	PD	38 d	NA
4	Özener İç [19]	1993	30	Suspected PE, placental abruption	PD	44 d**	NA**
5	Frimat M [20]	2016	32	PPH, PE	HD	210 d	eGFR 24
6			32	PPH, PE	HD	62 d	eGFR 35
7			39	PPH	HD	66 d	eGFR 46
8			34	PPH	HD	120 d	eGFR 18
9			34	PPH	HD	60 d	eGFR 46
10			40	PPH	HD	46 d	eGFR 49
11	Béji S [21]	2017	48	PE, placental abruption	HD	3 y	Re-initiated dialysis
12	Present case		32	PPH, PE, HELLP syndrome	PD	229 d^#^	sCr 2.67

eGFR expressed in mL/min/1.73m^2^; sCr expressed in mg/dL. sCr = serum creatinine; eGFR = estimated glomerular filtration rate; PE = pre-eclampsia; PPH = postpartum hemorrhage; HELLP = hemolysis, elevated liver enzymes, and low platelets; HD = hemodialysis; PD = peritoneal dialysis; d = days; y = years; NA = not available. *Recovered by day 43; dialysis was re-initiated 15 months later. **Case 4 received PD, successfully recovered from dialysis by day 44, and was discharged with a serum creatinine level of 3.2 mg/dL. The patient remained independent of dialysis with chronic kidney disease and was lost to follow-up ~ 1 year after discharge. ^#^The duration of dialysis in the present case included a short period of continuous hemodiafiltration followed by intermittent hemodialysis.
